# Preface

**Published:** 2009-05

**Authors:** Fred Saad

**Affiliations:** Division of Urology, Centre Hospitalier de l’Université de Montréal, University of Montreal, Montreal, QC

It was with pleasure that I accepted to serve as editor of this timely supplement dedicated to kidney cancer. In the twenty years that I have been involved in the management of this disease, it has been spectacular to witness the evolution in treatment options. Urologic oncology is truly a rags-to-riches story. Through research and collaboration between the various specialists involved in treating kidney cancer, the disease has moved from having radical nephrectomy as the only useful treatment available, to having an extensive array of options for localized disease at one end of the spectrum and novel therapeutic strategies for advanced renal cell carcinoma at the other. Kidney cancer has become a state-of-the-art example of therapy tailored to the individual patient and to the extent of the disease.

For the small renal mass, there is no longer any controversy about whether to spare the kidney and to remove only the area affected by cancer. Today, the choice is whether certain small tumours even need to be treated, depending on the patient’s life expectancy and on the tumour’s characteristics as assessed by imaging, pathology, and molecular criteria. In an explosion of effort, new ablative techniques are also being studied to further reduce morbidity in the treatment of small renal masses.

Pivotal research on the pathways related to kidney cancer causation and the molecular steps involved in its progression have led to new targeted therapies. In a very short time, these new agents have changed the landscape of oncology in a way that has rarely been seen in medicine. This transition has basically moved the field from drugs with very little activity in kidney cancer to five new therapeutic options that can prolong and improve the lives of patients. Because of the varying targets of the new drugs in the pathways that lead to proliferation and resistance, the era has arrived in which, through the judicious use of available options, patients can sometimes be offered first-, second-, third-, and even fourth-line treatment options.

Even more impressively, progress is still being made. Intense research is still ongoing. New therapeutics are on the horizon. And the roles of the already-introduced targeted therapies are also being determined, including how best to sequence targeted therapies and whether combined modalities will further improve outcomes.

I am extremely grateful to the experts who accepted and took the time to contribute to this project. All are world leaders in the field of kidney cancer, and they have provided insightful and practical illumination on the approaches to the management of this disease. Our community can be proud of the expertise here in Canada, given that many of this supplement’s authors have contributed significantly to the research that has led to confirmation of the efficacy of the new therapeutic options. Many have also been instrumental in the development of new guidelines for the management of kidney cancer. Their work has contributed to better care for patients with kidney cancer in Canada and around the world. I also thank the team at *Current Oncology* for their work in moving this project from idea to reality. Their expertise and professionalism were exemplary.

